# Assessing Comprehensive Spatial Ability and Specific Attributes Through Higher-Order LLM

**DOI:** 10.3390/jintelligence13100127

**Published:** 2025-10-05

**Authors:** Jujia Li, Kaiwen Man, Mehdi Rajeb, Andrew Krist, Joni M. Lakin

**Affiliations:** Department of Educational Studies in Psychology, Research Methodology and Counseling, College of Education, University of Alabama, Tuscaloosa, AL 35487, USA; kman@ua.edu (K.M.); mrajeb@ua.edu (M.R.); atkrist@crimson.ua.edu (A.K.); jlakin@ua.edu (J.M.L.)

**Keywords:** spatial reasoning ability, CHC, CDM

## Abstract

Spatial reasoning ability plays a critical role in predicting academic outcomes, particularly in STEM (science, technology, engineering, and mathematics) education. According to the Cattell–Horn–Carroll (CHC) theory of human intelligence, spatial reasoning is a general ability including various specific attributes. However, most spatial assessments focus on testing one specific spatial attribute or a limited set (e.g., visualization, rotation, etc.), rather than general spatial ability. To address this limitation, we created a mixed spatial test that includes mental rotation, object assembly, and isometric perception subtests to evaluate both general spatial ability and specific attributes. To understand the complex relationship between general spatial ability and mastery of specific attributes, we used a higher-order linear logistic model (HO-LLM), which is designed to simultaneously estimate high-order ability and sub-attributes. Additionally, this study compares four spatial ability classification frameworks using each to construct Q-matrices that define the relationships between test items and spatial reasoning attributes within the HO-LLM framework. Our findings indicate that HO-LLMs improve model fit and show distinct patterns of attribute mastery, highlighting which spatial attributes contribute most to general spatial ability. The results suggest that higher-order LLMs can offer a deeper and more interpretable assessment of spatial ability and support tailored training by identifying areas of strength and weakness in individual learners.

## 1. Introduction

Spatial ability has been widely recognized as an effective indicator for predicting individuals’ performance in visuospatial and related tasks, particularly within the fields of Science, Technology, Engineering, and Mathematics (STEM). It refers to an individual’s capacity to generate, retain, retrieve, and transform well-structured visual images (Lohman 1979, 2014). Recent research has argued that spatial ability is not a unitary entity, but a multidimensional cognitive domain composed of distinct but interrelated subcomponents (Carroll 1993). However, traditional spatial assessments usually focus narrowly on a single task type (e.g., mental rotation or objective assembly). These assessments can measure specific abilities precisely but lack the ability to measure general spatial ability.

To address this issue, this study introduces a mixed spatial test that includes three types of tasks: mental rotation, object assembly, and surface development. This study uses a higher-order linear logistic model (HO-LLM), a type of cognitive diagnostic model (CDM), to evaluate general spatial ability and specific attributes simultaneously. In the CDM framework, a solid theory is essential for defining a high-quality Q-matrix that links items to latent cognitive attributes and ensures the validity of the assessment. However, there is no consensus on the classification of spatial attributes, which prevents us from defining a perfect Q-matrix. The Cattell–Horn–Carroll (CHC) theory of intelligence offers a flexible framework for exploring and comparing diverse spatial attributes classifications. This study examines four theories of spatial ability attribute classification for constructing Q-matrices and compares their influence on model fit, parameter estimation, and diagnostic precision.

This approach aims to improve spatial assessment and enhance interpretable understanding of spatial reasoning ability by developing a comprehensive test and employing HO-LLM. In the following sections, we will introduce the theoretical framework, test design, modeling method, and research questions.

### 1.1. Structure of Intelligence

The Cattell–Horn–Carroll (CHC) theory of cognitive abilities offers a comprehensive taxonomy of intelligence, synthesizing insights from multiple overlapping cognition theories. This framework provides a common framework for intelligence researchers to communicate their findings (McGrew 2009). In this study, we will be exploring the structure of spatial ability under CHC theoretical framework. McGrew (2009) addressed CHC theory by integrating Cattell–Horn’s and Carroll’s frameworks, highlighting the relationship between specific, broad, and general factors of visual processing (Gv) and providing a comprehensive structure for examining the role of Gv in cognitive assessment and development.

**Figure 1:**
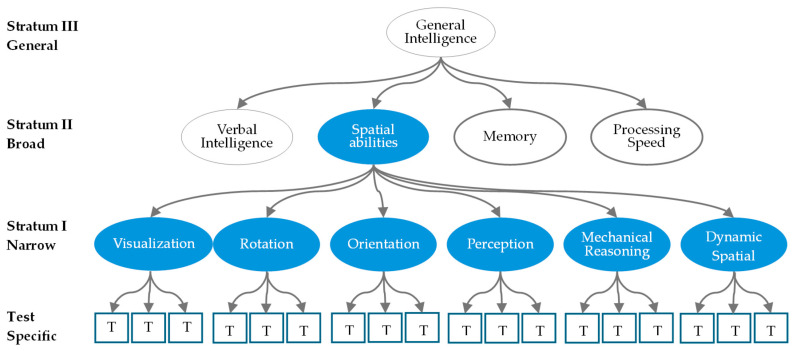
The Spatial Abilities in CHC framework with unidimensional item design.

In the CHC framework, intelligence is organized into three hierarchical strata (Figure 1), showing the relationships among general intelligence, broad abilities, and specific (narrow) abilities. General intelligence (g) sits at the apex of this framework, which captures the shared variance across all broad and narrow abilities. Broad Abilities (Stratum II) include abilities like Gv (Visual Spatial), Gf (Fluid Reasoning), and Gc (Crystallized Intelligence), and these abilities represent higher-level domains of cognition, emerging from various clusters of specific abilities. Specific Abilities (Stratum I) are narrower sub-abilities or attributes derived from broader abilities, often interconnected and exhibiting significant correlations with one another. In this study, we adopt a spatial ability framework with six specific spatial abilities proposed by Lakin (2024), as an example. Two alternative ability frameworks are also considered and compared in the following section. In this research, we focus on assessing spatial ability, with particular attention to the structures of Stratum I (specific abilities) and Stratum II (broad abilities).

Additionally, in traditional spatial test design, items for hierarchical structural models are typically constructed to assess only one specific ability or attribute, particularly in confirmatory factor analysis (CFA) contexts. As illustrated in Figure 1, the design assumes that each latent specific ability is measured by multiple distinct items.

### 1.2. Spatial Ability Classification

Although the CHC framework allows for flexibility in discussing and defining the classification of general intelligence and specific abilities or attributes, researchers have yet to reach a consensus regarding the classification of specific types of spatial ability. It is still a challenge to select a comprehensive framework that provides more precise structure of spatial ability and its underlying attributes. Table 1 presents a chronological overview of the most influential spatial ability classification frameworks for explaining the model selection in this study.

The systematical classification of spatial ability attributes originated from early psychometric work by Thurstone (1941). After that, McGee (1979), Linn and Petersen (1985), Lohman et al. (1987), and Carroll (1993) further developed their framework through factor analyses, primarily focusing on latent dimensions of spatial ability, such as visualization, orientation, and rotation.

Other research has focused on the cognitive processes needed for solving spatial tasks (Cooper and Shepard 1973). More recently, Hegarty et al. (2010) and Uttal et al. (2013) further incorporated dynamic spatial reasoning and perspective-taking into their process-driven framework. These changes shifted focus from structure to cognitive mechanisms.

Factor-based and process-driven classifications originate from different research backgrounds, provide varying diagnostic information, and exhibit some overlap. Lakin (2024) synthesized these models and proposed a more comprehensive framework that includes visualization, rotation, orientation, perception, mechanical reasoning, and dynamic spatial ability to broaden the theoretical coverage. This study also raises a new question about whether increased complexity might hinder model estimation and effectively offer nuanced diagnostic information. To answer this question, we ultimately select four spatial ability classifications with varying complexity (Linn and Petersen 1985; Hegarty et al. 2010; Uttal et al. 2013; Lakin 2024) to explore the trade-offs in model complexity and diagnostic value.

Based on previous categories and theories of spatial ability, Lakin (2024) addressed a comprehensive categorization, including visualization, rotations, orientation, perception, mechanical reasoning, and dynamic spatial ability.

### 1.3. Comprehensive Spatial Test

As previously mentioned, researchers have proposed various frameworks for Stratum II and Stratum III based on factor analysis, promoting the development of numerous tests to assess intelligence. These assessments typically include diverse item types, such as the Stanford-Binet Intelligence Scales-Fifth Edition (SB5; Roid 2012), the Wechsler Intelligence Scale for Children, Fifth Edition (WISC-V; Wechsler 2014), and the Reynolds Intellectual Assessment Scales, Second Edition (RIAS-2; Reynolds 2010).

Conversely, in past decades, researchers and educators have developed various spatial ability tests to assess specific aspects of spatial ability (Campos and Campos-Juanatey 2020; Hegarty 2004; Kozhevnikov and Hegarty 2001; Mitolo et al. 2015; Shepard and Feng 1972; Tragantzopoulou and Giannouli 2024; Workman and Lee 2004). These tests are well-designed and have been shown to provide valid and reliable assessments of specific spatial ability attributes, aligning with Stratum I in the CHC framework.

In this study, we aim to bridge the gap between the comprehensive evaluation of spatial ability and its specific attributes with a comprehensive spatial test, integrating appropriate types of specific tests, such as Mental Rotation (MR), Object Assembly (OA), Surface Development (SD), among others. Given the need for a more complete assessment of spatial ability, this research further aims to develop a comprehensive spatial test that integrates multiple types of tests. This approach will ensure that specific spatial ability is adequately measured, allowing for a more holistic understanding of individuals’ spatial competencies and abilities.

### 1.4. Traditional Methods of Modeling Hierarchical Structure of Intelligence

Classical models like IRT and CTT are effective for assessing latent traits but often assume unidimensionality, limiting their ability to capture the complex and multidimensional nature of item responses. Specifically, CTT provides test-level reliability estimates and evaluates overall test performance, while IRT focuses on item-level characteristics, but does not explicitly model the relationships between broader and specific abilities. Generally, these models assume that each item measures only one specific dimension of ability, which limits their ability to capture the multidimensional nature of item responses.

Hierarchical models go beyond unidimensional models by capturing the complexity of cognitive abilities. Hierarchical confirmatory factor analysis (CFA) model latent variables based on item-level covariance, estimating a first layer of specific abilities, and then using the covariance of the first level abilities to estimate a second-order model. In traditional CFA, each item is typically modeled as being affected by only one latent variable (a single factor loading onto the item) or, in some cases, a secondary higher-order factor in hierarchical CFA. This structure assumes a unidimensional or nested multidimensional framework, which limits its ability to account for more complex relationships between items and multiple cognitive processes. Bifactor models, also grounded in factor analysis, specify two independent sets of factors: a general factor directly from item-level covariance, and specific abilities modeled as orthogonal residual factors that capture unique variance beyond the general factor. For example, specific abilities may be specified as well as a latent general factor (Lakin and Gambrell 2014). While this adds flexibility compared to CFA, it still restricts each item to being influenced by one general and one specific factor, making them less suitable for modeling tasks requiring a combination of multiple attributes.

To address the limitations of previous models, Kovacs and Conway (2016) recently addressed a novel concept called Process Overlap Theory (POT) to offer an alternative option for previous models. POT suggests that the positive manifold (the connections between cognitive activities) comes from overlapping cognitive processes or specific attributes, rather than from a single general ability or restricted hierarchical structure. In the POT framework, general spatial ability is not a single latent trait but rather a statistical reflection of the processes engaged across spatial tasks. The argument recommends that researchers use multidimensional modeling methods, which are ideal for assessing complex spatial tasks requiring multiple attributes.

### 1.5. Higher-Order LLM

Traditional measurement models (e.g., IRT, CTT) are limited in their capacity to provide a granular understanding of specific abilities or to capture nuanced interactions between cognitive processes. By treating specific abilities as either dependent on or separate from the general factor, these models may overlook true interplay of cognitive attributes, especially for complex domains like spatial reasoning or problem-solving. As a result, there is a growing need for models that can provide more detailed insights into specific cognitive abilities and their relationships without being overly constrained by hierarchical or orthogonal assumptions.

De La Torre (2011) addressed that CDMs are specifically designed to handle scenarios where an item is influenced by multiple latent variables (i.e., attributes). Certain CDMs are capable of modeling the interaction among multiple attributes required to correctly respond to an item, such as skills, knowledge components, or cognitive processes. This makes CDMs useful for assessments where tasks involve multiple skills, as they inform us on how different attributes interact and affect performance. Furthermore, CDMs provide deeper diagnostic insights by allowing each item to assess multiple specific abilities or attributes simultaneously. This feature is particularly relevant for spatial tests, which are often designed to assess specific tasks or professional requirements while still involving multiple spatial components. By using a CDM approach, we can better understand the interaction between different spatial abilities within each test item, leading to more precise diagnostic information and personalized feedback.

We can use different CDMs depending on the application of the research. In this research, we will utilize HO-LLM. We use this model because (1) we assume general spatial ability could impact specific spatial ability attributes, and (2) through a pilot study on comparing multiple CDMs’ model fit, the HO-LLM outperformed other CDMs. The results of this pilot study are described in the results section of this manuscript.

The HO-LLM is a parsimonious model for the joint distribution of high-dimensional attribute vectors, linking specific cognitive skills to a broadly defined latent trait, similar to the *θ* in item response models (De La Torre and Douglas 2004). It effectively models the relationship between general ability (like general spatial ability) and specific knowledge/traits (specific spatial ability attributes), providing a simple yet informative framework for cognitive assessment.

The probability of mastering attribute for individuals is defined as

(1) $$P\left(\alpha_{k}=1|\theta_{i},\lambda_{0k},\lambda_{1k}\right)=\frac{\exp(\lambda_{1k}\theta_{i}+\lambda_{0k})}{1+\exp(\lambda_{1k}\theta_{i}+\lambda_{0k})}$$

in which, $\alpha_{k}$ represents the *k*th specific attribute; θ is the latent trait or general ability of individual *i*; $\lambda_{0k}$ is intercept parameter that reflects the baseline difficulty of the attribute; $\lambda_{1k}$ represents the discrimination, indicating how sensitive the attribute mastery probability is to changes in the latent trait. The probability of joint attributes can be written as

(2) $$P\left(\alpha |\theta_{i},\lambda \right)=\prod_{k}P\left(\alpha_{k}|\theta_{i},\lambda \right)$$

in which, α is vector of attributes representing the mastery of each specific attribute ($\alpha_{k}$); **λ** represents model parameters that influence the probability of mastering each attribute; $\prod_{k}$ is the product over all attributes, indicating that the joint probability is the product of individual attribute probabilities.

### 1.6. Task-Type-Level Q-Matrix

Q-matrices are typically defined for test-items based on various skills required for successfully answering the item. In this scenario, the focus is on defining mastery of key skills. Another way to look at Q-matrices is that they enable capturing shared cognitive processes across multiple items within the same task category. This approach assumes that items belonging to the same task type (e.g., arithmetic vs. geometry) rely on similar skills or cognitive attributes, which enhances consistency and interpretability of the latent traits.

Developing a Q-matrix for a learning progression of skill mastery is complex and usually relies on subject matter experts to generate a complex and extended set of defined attributes (Embretson 2010; Ivie and Embretson 2010). In contrast, a simplified Q-matrix at the task-type level defined the relationship between item and attributes based on task types (e.g., mental rotation, object assembly). Meanwhile, task-type Q-matrices simplify model construction by reducing the number of parameters, allowing for more efficient modeling, particularly when working with multidimensional CDMs.

### 1.7. Research Questions

1. How effectively can Higher-order LLM estimate a comprehensive general spatial ability through a mixed spatial test?
2. Does a Q-matrix with a more detailed spatial attribute classification improve model fit and classification accuracy compared to a simplified Q-matrix?

## 2. Materials and Methods

### 2.1. Spatial Reasoning Tasks

In this research, we used two widely used spatial reasoning tasks, mental rotation test (MRT) and objective assembly (OA), as well as a lesser-used test called isometric perception (IP). The combination of three types of tasks extensively covers most sub-spatial attributes, offering a comprehensive spatial reasoning assessment.

**Figure 2:**
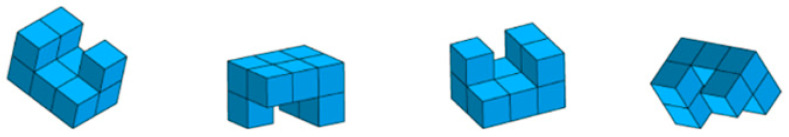
An Example Item from the Mental Rotation Task. This task presents a 3D target object (far left) and a set of alternative objects. Examinees are asked to identify which of the remaining options is a rotated version of the target object. The third option (far right) is the correct one.

The first type of spatial task is called the mental rotation test, which is widely used for measuring spatial ability, especially for testing the capacity to mentally manipulate three-dimensional objects. Vandenberg and Kuse (1978) originally developed the MRT. In our research, each MR task presents four rotated alternatives, requiring examinees to identify the one figure that mismatches the other three after mental rotation (see Figure 2). The challenges may include mirrored versions, incorrect orientations, or cube placement. Performance on spatial reasoning tasks has been shown strongly associated with performance in fields such as engineering, architecture, and surgery, where professionals must mentally manipulate 3D objects (e.g., Shepard and Metzler 1971; Uttal et al. 2013).

**Figure 3:**
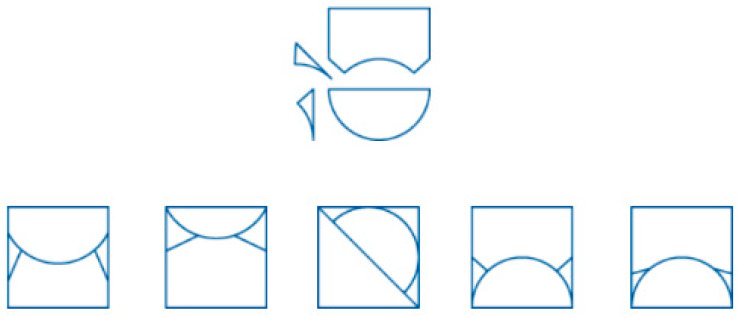
An Example Item from the Object Assembly Task. The top panel shows disassembled visual pieces of an object, possibly including curved, straight edge, and regular pieces (e.g., semicircle, rectangle, etc.). It requires examinees to determine which of the five answer choices below accurately represents the correctly assembled object. The fourth option is the correct one.

The second type of task is object assembly, also known as the Minnesota Paper Form Board Test (MPFBT) (Quasha and Likert 1937), which is a widely used assessment of visualization skills. This test requires examinees to mentally assemble given pieces to form a complete object, shown as Figure 3. The U.S. military commonly employs the OA test as a subtest of the ASVAB for enlistment and placement. OA tasks primarily measure two-dimensional rotation and visualization skills, classifying them as formboard tasks (Eliot and Smith 1983; Lohman 1979). Incorrect options may include misleading configurations such as misaligned curves, incorrect rotation, or geometrically incompatible combinations. OA tasks require the mental construction of complex wholes from parts, are relevant to mechanical reasoning and hands-on technical skills, especially for manufacturing, engineering, and even spatial problem solving in everyday tool use (e.g., Lohman 1996; Carroll 1993).

**Figure 4:**
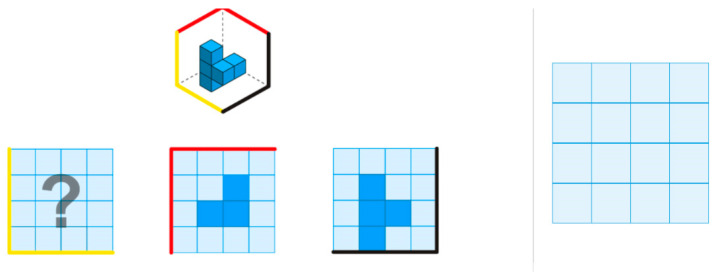
An Example Item from the Isometric Perspective Task. The top-left panel shows a 3D object in an isometric view composed of stacked cubes. In the bottom-left panel, the red- and black-edged grids represent the object’s top and right orthographic views. Examinees are required to mentally rotate or visualize the object to infer the correct left view and reproduce it using the empty grid in the panel to the right of gray line.

The third task type in our mixed test is isometric perspective (IP), which presents three-dimensional objects in an isometric view and requires examinees to mentally manipulate the structure to infer or construct corresponding orthographic projections (Carroll 1993; Eliot and Smith 1983). Figure 4 is an example of an IP task, requiring examinees to infer the missing orthographic projection by analyzing the isometric view and the provided top and right views. Misjudging depth, cube positions, height, and layout information across views often leads to incorrect responses. IP tasks reflect real-world challenges such as interpreting engineering blueprints or using CAD software (e.g., AutoCAD and SolidWorks), such as industrial design, architecture, and spatial planning (e.g., Eliot and Smith 1983; Carroll 1993).

Three spatial task types included in this study were originally developed for adults or the military (like the ASVAB). For this study, all the task content was modified to ensure children (ages 9–14) might complete them independently. These adaptations included simpler visual stimuli, age-appropriate instructions, no time limits, and explicit feedback, with standardized administration ensuring participants understood the tasks before beginning.

Previous research addressed that spatial abilities such as mental rotation, visualization, and object assembly develop rapidly during middle childhood, with substantial growth between ages 6–10. Mental rotation improves rapidly from ages 6–8, while extrinsic spatial skills and navigation using cognitive maps advance more between ages 8–10 (Hawes et al. 2015; Wimmer et al. 2017; Hodgkiss et al. 2021; Burles et al. 2020). These findings also support the fact that tasks in our study are appropriate for samples of 9- to 14-year-olds.

### 2.2. Definition of Q-Matrices

In this study, we use HO-LLM, one type of CDM to offer diagnostic information by identifying which spatial ability attribute an individual has mastered. In CDM framework, a well-defined Q-matrix, which maps items to target attributes, is the critical factor for achieving a valid and reliable estimation. Briefly, each entry indicates whether a specific skill or attribute is needed to answer a given item correctly, denoted by 0 or 1. The Q-vector is part of Q-matrix (representing the relationship between individual items and attributes) is defined at the test level rather than the item level. This approach reflects the assumption that all items within a task type (e.g., mental rotation) assess the same set of spatial attributes. It also helps reduce subjective bias in expert-defined Q-matrices by simplifying and standardizing attribute assignment across similar items.

This research started by defining four Q-matrices following the previously discussed categories and theories of spatial abilities (see Table 2, Table 3, Table 4 and Table 5). In these four Q-matrices, this approach means that for each spatial task type (e.g., Mental Rotation Test), we assign a single Q-vector that applies to all items belonging to that specific task type.

### 2.3. Data

We collected spatial test data from *N* = 181 students in grades 4–8 (ages 9–14), and *N* = 122 participants completed all three types of spatial tasks, including the Mental Rotation Test (MR), Object Assembly (OA), and Isometric Perception (IP), separately. Each subtest requires participants to finish in one hour, initiated by a visual example. These examples ensured participants understood the task requirements before beginning the scored items, helping to reduce confusion and promote fairness. The tasks were chosen to represent a developmental range appropriate for assessing spatial reasoning abilities, given that these skills develop and diversify significantly during childhood and early adolescence.

All data collection procedures were guided by our approved IRB protocol. All participants were recruited through local schools and community outreach programs, with the assistance of parents and educators who facilitated initial contact. Retention strategies included providing clear instructions, ensuring a comfortable testing environment, and minimizing session durations to avoid fatigue. Participants received a small gift card in return for their participation. To protect participants’ confidentiality all collected data was anonymized, with participants assigned unique ID numbers to protect personal information.

### 2.4. Analysis Plan

We used the GDINA version 2.9.12 (Ma and de La Torre 2020b) R package, which supports a variety of CDMs including DINA, GDINA, LLM, and higher-order extensions, to conduct model estimation and comparison. We began our study by selecting a suitable CDM from eight options (DINA, DINO, GDINA, ACDM, LLM, RRUM, high-order GDINA, and high-order LLM) and comparing their model fit. Although we have created four Q-matrices, to avoid redundancy, we only presented the results of the model comparison using 6-attribute Q-matrices (based on Lakin’s spatial ability classification). Next, we will analyze item parameters (e.g., higher-order Theta, guessing, and slipping) and classification accuracy for each model. Finally, we will use the person parameter to address how four Q-matrices can offer different insights on individuals’ spatial ability structure.

## 3. Results

### 3.1. Model Fit and Comparison

We chose a high-order LLM because it simultaneously estimates a general latent ability and specific attributes. We also conducted a pilot study to compare higher-order LLM to other CDMs and explore its efficiency in spatial ability assessment. To simplify the model-selection process, we only compared all eight CDMs using the more informative six-attribute Q-matrix (based on Lakin’s spatial ability classification). Given that only DINA, DINO, and GDINA are nested models, Table 6 only shows relative model fit indices to ensure a comprehensive comparison across all models, including AIC, BIC, CAIC, and SABIC. Among these indices, the Akaike Information Criterion (AIC) is the most prevalent and basic index, helping select model by balancing complexity and fit. Meanwhile, the Bayesian Information Criterion (BIC) imposes a heavier penalty on model complexity. The Consistent Akaike Information Criterion (CAIC) is stricter than BIC, preferring simpler models as the sample size increases. The Sample-Size Adjusted BIC (SABIC) enhances dealing with small sample sizes. For all four indices, lower values indicate better model fit. After comparing model fit indices of all eight CDMs, the high-order LLM model (HO-LLM) provides the best relative fit based on lowest AIC (4744.01), BIC (5316.03), CAIC (5520.03) and SABIC (4671.03), leading us to select it in our research.

In Table 7, the model with Lakin’s Q-matrix (Q_LH_) provides competitive relative fit based on AIC (4744.01) and SABIC (4671.03), while Linn’s Q-matrix (Q_LP_) has BIC (5179.74) and CAIC (5307.74). It means that both Lakin’s and Linn’s models balance model fit and complexity well. For absolute model fit, Hegarty’s Q-matrices (Q_H_) have the lowest RMSEA2 (0.046), indicating the best absolute fit among the models. RMSEA values closer to 0 indicate better fit; values below 0.05 are considered good. Meanwhile, Lakin’s has the lowest SRMSR (0.085) among the models but is still slightly above the recommended threshold of 0.08, indicating some minor model misfit.

Table 7 also includes classification accuracy, referring to how accurately the CDM can identify an individual’s mastery or non-mastery of specific skills or attributes. Linn’s Q-matrix achieves a best classification accuracy (0.931), which means it has identified a mastery pattern with 95.7% accuracy, an impressive value. Meanwhile, Lakin’s Q-matrix has the lowest CA (0.708), but values above 0.70 are still often considered acceptable in educational or psychological assessments. The reason could be that Linn’s Q-matrix only has three attributes, which can make it easy to detect attribute mastery.

Based on Table 7, we cannot simply decide which Q matrix is the exclusive optimization. They had a similar performance on model fit and classification accuracy, suggesting that model selection should consider the specific context and purpose of the analysis.

### 3.2. Item Fit

**Figure 5:**
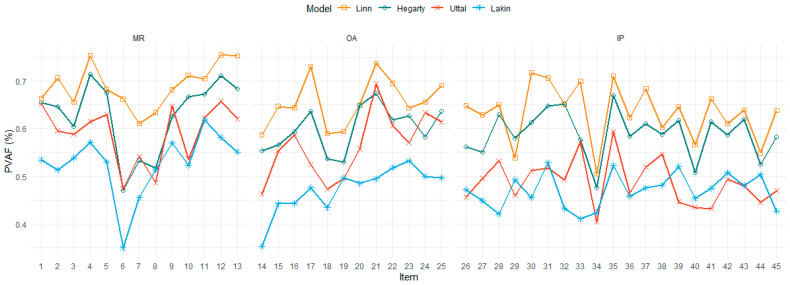
Item-level PVAF Comparison across Models (Linn, 1985; Hegarty, 2010; Uttal, 2013; Lakin, 2024) and Tasks. MR = Mental Rotation; OA = Objective Assembly; IP = Isometric Perception.

In CDM framework, Proportion of Variance Accounted For (PVAF) reflects the proportion of the total variability in the data that is explained by the model. Item-level PVAF can offer more details about how well the model accounts for the variance in responses to each specific item, supporting us make decisions on filtering items. In Figure 5, Linn’s model consistently outperforms others across all task types, standing around 0.7, indicating that it better captures the variance in the data. Hegarty’s model shows competitive performance, particularly in the MR item type. According to item-level PVAF, some items (e.g., 6, 14) show significantly lower PVAF (lower than 0.4) across all models, suggesting item misfit or modeling issues. The findings might indicate that these items are not well represented by the Q-matrix or are particularly challenging or ambiguous. Additionally, all models capture mental rotation tasks well, with the MR items exhibiting relatively high and stable PVAF. The OA and IP items show more fluctuation and variability, suggesting that item-specific dips need further investigation.

**Figure 6:**
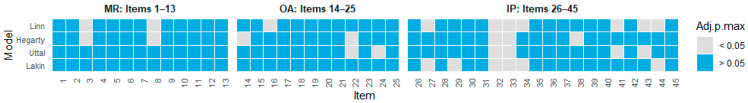
Adjusted *p*-Values for Max Correlation Across Models by Item Type.

We applied Bonferroni correction approach for calculating the maximum adjusted correlation (Adj.p.max) between each item and all other items (Bonferroni 1936) to identify potential item redundancy or model misfit. In Figure 6, the heatmap shows the adjusted *p*-values for each item across four models, listed by rows (Linn, Hegarty, Uttal, and Lakin). Items with Adj.p.max less than 0.05 indicate significant correlation with other items, which is a signal of potential misfit (shown in light grey). Items with Adj.p.max greater than 0.05 suggest an acceptable fit (shown in blue).

Across models, most items exhibit acceptable fit (blue), especially for the MR and OA tasks. However, there are notably problematic items concentrated in the IP tasks (Items 26–45). Specifically, items 32 and 33 consistently misfit across all models, which means that these items may be redundant or underrepresent this subset. Among four models, the Lakin model shows the best performance in MR and OA tasks, with fewer misfitting IP items. Overall, the heatmap of adjusted *p*-value identifies which items require further review and highlights the Lakin model relatively overperform other models in fitting diverse spatial item types.

In the HO-LLM framework, guessing and slipping parameters play a crucial role in interpreting unpredictable student performance. The guessing parameter indicates the likelihood that a participant without the necessary skill can still answer an item correctly through guessing. A high guessing parameter suggests that some items are vulnerable to random guessing or that these items may depend on additional mental resources or unmeasured attributes. The slipping parameter represents the probability that a student who has the required skill answers the item incorrectly. A high slipping rate indicates that certain factors (such as carelessness, misunderstanding, or fatigue) are leading to unexpected errors. We interpret items with high guessing or slipping as potentially problematic for diagnostic accuracy (De La Torre et al. 2010). However, there is not a fixed threshold to filter guessing or slipping parameter. In most cases, less than 0.3 are an acceptable value (Cuhadar and Salih 2022).

**Figure 7:**
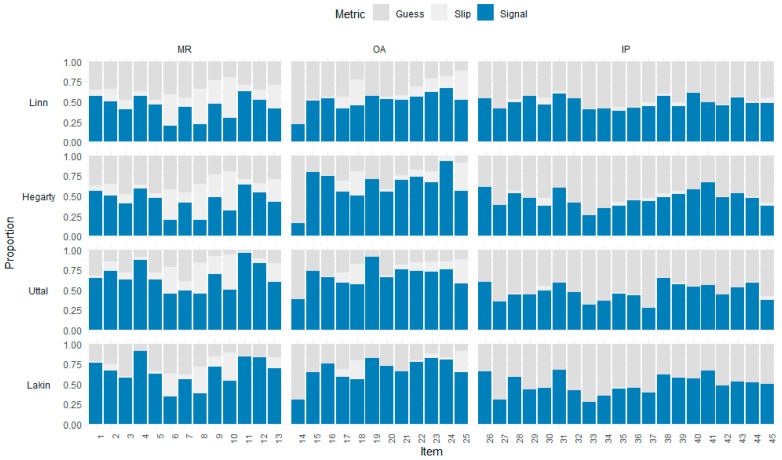
Stacked Column Plot of Slipping, Guessing, and Tested Rates by Model and Item Type. MR = Mental Rotation; OA = Object Assembly; IP = Isometric Perception. Guess = guessing parameter (top segment, grey); Slip = slipping parameter (middle segment, light grey); Signal = the true detectable mastery probability after trimming guessing and slip (bottom segment, blue).

In Figure 7, the Uttal and Lakin models show lower rates of guessing and slipping and higher rates of useful signals for most tasks, especially MR and OA tasks. Linn model has better rates of useful signals for some IP tasks. Overall, the Uttal and Lakin model may exhibit more information on person ability estimation without the impact of slipping and guessing.

Regarding task type, the OA task showed the best performance with higher signal section (shown as blue) and smaller guessing and slipping parameters (gray and light grey, respectively), except item 14. Some items of MR task have higher guessing parameters, such as item 6, 8, and 19, indicating some participants with higher ability could answer incorrectly. The IP task type exhibits lower slipping parameters, but higher guessing parameters compared to the other two task types. This suggests that while children who understand the task tend to answer correctly without errors, others may guess, indicating that this type may be particularly challenging for younger examinees.

### 3.3. Person Parameters

Higher-order LLM simultaneously estimates higher-order latent ability and attribute mastery probabilities. We created an Ability-Mastery Alignment Plot (AMAP) to visualize the relationship between higher-order latent ability (also known as theta, shown in top section) and mastery pattern profiles (shown in bottom section) derived from CDMs. For example, in Figure 4, the top section displays higher-order ability estimates, reflecting the general spatial ability level of each person, in which each dot represents an individual. The bottom section shows mastery pattern profiles across multiple attributes (e.g., spatial visualization (SV), mental rotation (MR), and spatial perception (SP), in which “×” markers indicate mastery (1) or blank markers for non-mastery (0) for each attribute. Combining the top and bottom sections, this plot indicates how individuals’ theta aligns with their mastery profile.

**Figure 8:**
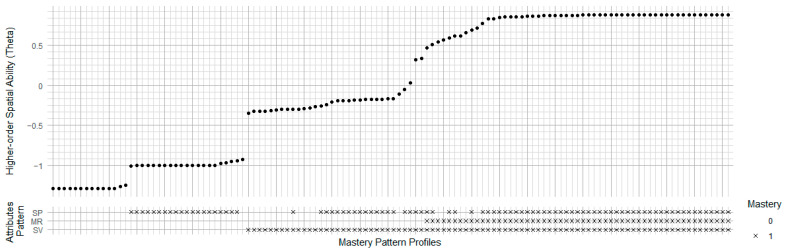
Ability-Mastery Alignment Plot of Linn Model. Theta = Higher-order Spatial Ability; SP = Spatial Perception; MR = Mental Rotation; SV = Spatial Visualization.

Figure 8 shows the relationship between higher-order spatial ability (theta) and attribute mastery patterns in the Linn model. Specifically, the rightmost points in the top plot have greater theta values (around 0.95), while these individuals have full mastery across all three attributes (SP, MR, and SV) in the bottom plot. Participants without mastering mental rotation attribute (mastery pattern (1,0,1)) have a much lower theta than those who master all attributes (mastery pattern (1,1,1)), dropping from 0.95 to −0.20. This highlights that mental rotation is the most critical attribute for higher-order spatial ability. One possible reason is that mental rotation may involve greater executive demands, such as transformation and spatial updating, compared SV, which only requires recognition or static mental imaging. When spatial perception is absent (mastery pattern (0,0,1)), the higher-order ability only slightly drops from −0.20 to −0.25, suggesting that spatial perception has a minor impact. Moreover, only mastering spatial perception (mastery pattern (1,0,0)) results in a low spatial ability of around −1.0, which is just slightly higher than non-mastery of any attributes (mastery pattern (0,0,0)). This finding indicates that SP alone does not substantially support higher spatial ability in Linn’s model. In conclusion, the results suggest that in Linn’s spatial ability framework, mental rotation is the most important attribute for supporting high spatial ability, while spatial perception plays a less significant role.

**Figure 9:**
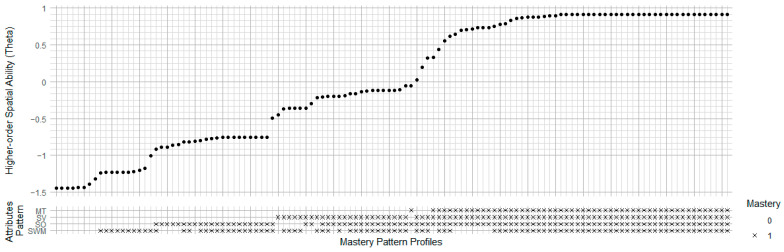
Ability-Mastery Alignment Plot of Hegarty Model. MT = Mental Transformation; SV = Spatial Visualization; SO = Spatial Orientation; SWM = Spatial Working Memory.

Figure 9 shows the relationship between higher-order spatial abilities and attribute mastery patterns in the Hegarty model. Like the Linn model, the rightmost points in the top plot also have the highest theta values (around 0.95) with full mastery across all four attributes (MT, SV, SO, and SWM) in the bottom plot. Theta decreases progressively with the absence of attributes, following the order of MT, SV, SO, and SWM. Notably, the absence of spatial working memory (SWM) (mastery pattern (1,1,1,0)) has only a slight impact on general ability (around 0.8), whereas the absence of mental transformation (MT) causes a substantial decrease in ability (around −0.2). This pattern indicates that while mastering mental transformation attributes is comparatively challenging for individuals, it is also the key element for achieving the highest general spatial ability.

**Figure 10:**
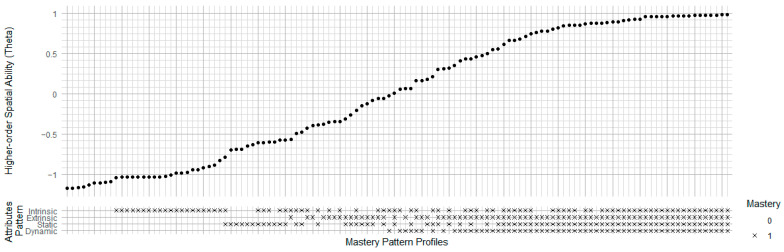
Ability-Mastery Alignment Plot of Uttal Model. Intrinsic: Spatial processing that focuses on the internal configuration of an object. 2 Extrinsic: Spatial processing involving relations between objects or between an object and its environment. 3 Static: Involves processing fixed spatial information with no transformation. 4 Dynamic: Involves spatial transformation or mental manipulation of objects in space.

Figure 10 shows the relationship between higher-order spatial abilities and attribute mastery patterns in the Uttal model. Like the previous Linn model and Hegarty model, the rightmost points in the top plot also have the highest theta values (around 0.95) with full mastery across all four attributes (intrinsic, extrinsic, static, and dynamic) in the bottom plot. The Uttal model’s theta has a more linear trend than the previous model’s clear step-like structure. This indicates that the combination of attributes has a nuanced effect on theta. Dynamic spatial ability is the most important attribute, yet only a small percentage of individuals master it, whereas intrinsic spatial ability plays a minor role, with the majority mastering it. Generally, to improve students’ general spatial ability, enhancing training on dynamic ability is an effective method.

**Figure 11:**
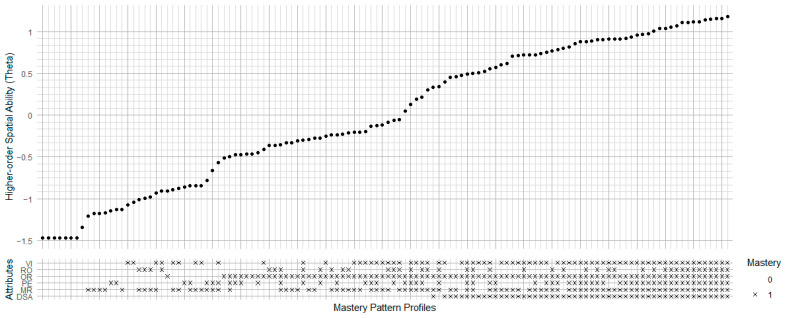
Ability-Mastery Alignment Plot of Lakin Model. VI = Visualization; RO = Rotation; OR = Orientation; PE = Perception; MR = Mechanical Reasoning; DSA = Dynamic Spatial Ability.

In Figure 11, the rightmost points in the top section, representing the highest theta values, align with full mastery patterns across all six attributes (VI, RO, OR, PE, MR, and DSA) in the bottom section. Most participants consistently master OR (Orientation) and MR (Mechanical Reasoning), indicating that mastery of these two spatial ability attributes is comparatively easier than others. From the highest theta, we found that the first missing attribute is Ro (Rotation), indicating the difficulty of mastering Ro and its major impact on general spatial ability. This finding provides the same result in the Linn model, which also includes the mental rotation attribute.

In conclusion, the analyses across Figure 8, Figure 9, Figure 10 and Figure 11 reveal distinct patterns in how various spatial attributes contribute to general spatial reasoning ability (theta) within different spatial ability classification frameworks. Across all models and framework, we found that certain attributes emerge as critical to higher general spatial reasoning ability, such as mental rotation in the Linn model, mental transformation in the Hegarty model, dynamic spatial processing in the Uttal model, and mental rotation in the Lakin model. These attributes likely serve as core components in fostering a robust spatial skillset, regardless of the specific classification framework.

## 4. Discussion

### 4.1. Major Findings and Contributions

A major part of this study is creating and using a comprehensive spatial reasoning assessment that incorporates multiple task types—Mental Rotation (MR), Object Assembly (OA), and Isometric Perspective (IP). Unlike traditional spatial tests that often rely on a single task type (e.g., the Mental Rotation Test), which may only measure a narrow band of spatial ability (such as visualization or transformation), our multi-type design enables the measurement of a broader range of spatial attributes simultaneously. This approach ensures that general spatial ability is not just determined by performance on a single task type, which could overemphasize one or more certain spatial attributes and distort estimation on general spatial ability. A well-designed comprehensive spatial reasoning assessment can offer a more valid and representative assessment of spatial reasoning.

Another major contribution is that the HO-LLM model can handle multidimensional test structure, which is a challenge for most psychometric models based on the assumption of unidimensionality. Additionally, HO-LLM is a more ideal model for spatial ability assessment than other CDMs. It estimates individuals’ general spatial ability and attribute mastery patterns simultaneously, highlighting the two-level structure of spatial ability and the importance of each attribute in solving spatial reasoning tasks. In our study, we applied this model across three prevalent spatial ability classifications (Linn and Petersen 1985; Hegarty et al. 2010; Uttal et al. 2013) and a newly developed one (Lakin 2024), identifying the most important attributes in each framework. The results show three distinct critical spatial attributes: mental rotation in both the Linn and Lakin models, mental transformation in the Hegarty model, and dynamic spatial processing in the Uttal model. These findings can guide pedagogical design and educational interventions aimed at enhancing spatial reasoning by focusing on critical spatial ability attributes.

The third finding is the flexibility of the CHC theoretical framework. Based on the CHC theoretical framework, our research used four spatial ability classifications to create Q-matrices for HO-LLM. After comparing model fit, classification accuracy, item fit, and both item and person parameter estimation, we found that the number and nature of attributes in the Q-matrix substantially impacted diagnostic outcomes. However, a theoretically justified spatial ability classification and a well-defined Q-matrix can provide comprehensive and multidimensional insights into spatial ability assessment.

The last contribution is the Ability-Mastery Alignment Plot (AMAP), which can help researchers and educators to visualize and interpret the relationship between general spatial ability (theta) and attribute mastery patterns. Unlike traditional CDM research summaries that just focus on showing mastery patterns, the AMAP provides a clear way to understand how mastering specific spatial skills helps improve overall spatial ability for both individuals and groups. This visual tool advances cognitive diagnosis research by enhancing the transparency and utility of multidimensional profiles for both researchers and practitioners.

### 4.2. Alignment and Divergence with Prior Research

Our findings align with prior research that emphasizes mental rotation as a critical component of spatial reasoning (Shepard and Metzler 1971; Linn and Petersen 1985; Hegarty 2004). This study extended their findings by showing that mental rotation consistently emerged as the most influential attribute across multiple frameworks, including the Linn and Lakin models, which are the only two models that consisted of mental rotation attributes. This investigation reinforces the established view that mental rotation is a fundamental predictor of spatial ability (Uttal et al. 2013; Yildiz and Ahmet 2017). Furthermore, our results align with Hegarty’s (2004) idea that mental transformation is critical in spatial cognition, as we saw a substantial drop in general spatial ability when mental transformation attributes were absent. Our study also diverges from previous research by examining the role of dynamic spatial ability (Uttal et al. 2013). Unlike traditional assessments primarily focus on static spatial ability, our model shows that dynamic spatial ability also significantly contributes to higher-order spatial reasoning. This finding aligns with the emphasis of Uttal et al. (2013) on the trainability of dynamic spatial skills as essential components of spatial intelligence.

In contrast to earlier studies that predominantly used item-level Q-matrices (Li et al. 2025; Ouyang et al. 2023; Wang et al. 2018), we adopted task-level Q-matrices based on the assumption that spatial tasks often share overlapping cognitive demands. This approach reduces model complexity and enhances interpretability, which are two major limitations in classical CDM research.

Finally, this study adds to the discussion about model selection and evaluation by comparing multiple CDMs and their fit indices, including AIC, BIC, CAIC, and SABIC. Previous studies (De La Torre and Minchen 2014; Lenzen et al. 2007) emphasized the importance of selecting the optimal CDM for diagnostic purposes. Our results indicate that the HO-LLM model not only provides the best model fit but also effectively captures both general ability and specific mastery patterns, reporting fine-grained diagnostic insight in spatial ability assessment.

### 4.3. Implications

The major implication of this study is that the HO-LLM offers an effective and robust approach for simultaneously estimating general spatial ability and specific mastery patterns under the CHC intelligence framework. These diagnostic outputs can help create targeted training programs by focusing on essential spatial attributes, such as mental rotation, mental transformation, and dynamic processing, that mostly affect higher-order ability. Based on these findings, educators can prioritize certain curricular activities, such as 3D object manipulation, physical paper folding, or dynamic computer-based simulations, to cultivate these high-impact attributes.

For instructional developers, the multi-task-type spatial test (integrating MR, OA, and IP) is a thorough diagnostic tool to identify examinees across all specific spatial attributes, rather than just one or a few. The complete coverage of general and specific ability diagnoses ensures a reliable design of training plans, helping instructional developers focus on the most critical or underdeveloped attributes. For example, instructional developers can enhance spatial visualization exercises for students who struggle with mental rotation by involving object manipulation and multi-angle perspective.

The Ability-Mastery Alignment Plot (AMAP) is a novel visual tool developed to enhance interpretability by aligning individuals’ general spatial ability with mastery profiles across spatial attributes. AMAP enables teachers to diagnose both individual and group patterns of strength and weakness. This facilitates targeted instruction—for example, grouping students who show low mastery in spatial visualization or transformation for focused practice. AMAP allows teachers to diagnose both individual and group patterns of strength and weakness. This facilitates tailored instruction. For example, teachers can group students according to mastery pattern of spatial attributes to select focused practice for each group, efficiently strengthening underdeveloped abilities.

For assessment practitioners, the HO-LLM framework can support the development of the adaptive spatial testing systems by dynamically assigning tasks based on examinees’ mastery profiles. This allows for a more accurate measurement of general and specific spatial abilities by focusing on tasks that test the examinees’ weak abilities. The HO-LLM also detects unexpectedly high guessing or slipping issues and guides the adaptive system to avoid those items.

### 4.4. Limitations and Future Research

Despite these promising findings, this study still has limitations and space to improve. First, this study has a relatively small sample (N = 122), and the limited age range (ages 9–14) restricts generalizability across populations. According to Holden and Tanenbaum (2023), modern considerations for assessing human intelligence must focus on equity and fairness. Unfortunately, in this study, the small sample size and incomplete demographic information prevented us from examining differential performance across groups such as gender, age, or race/ethnicity, a limitation of validating the spatial ability classification models and the HO-LLM approach.

Second, simplified task-level Q-matrix design may overlook item-specific nuances, potentially reducing diagnostic precision. Keeping the same Q-vector (the relationship between item and attributes) at the task level improves interpretability and reduces complexity, but it may mask important cognitive variations within items. Future research could use data-driven methods, such as iterative refinement based on PVAF (Ma and de La Torre 2020a), to adjust the Q-matrix at the item level.

Third, although selecting different spatial ability classifications to create a Q-matrix can affect model fit and accuracy, all four frameworks provide unique multidimensional insights to meet different research interests. All frameworks consistently highlighted dynamic spatial ability, such as mental rotation and transformation, which is a key predictor of higher-order spatial reasoning ability. However, we still found high guessing (g > 0.3–0.4) and slipping (s > 0.2–0.3) parameters in certain items, indicating that low spatial ability still manages to get the item correct or high spatial ability may miss the correct answer. There are two potential reasons. One is the item relying on non-spatial cues (e.g., visual salience, heuristics). The other is that the item targets a narrow ability not fully covered in the Q-matrix, especially if the item requires executive processes (e.g., spatial working memory or attentional control). These potential abilities could be part of general g (part of CHC Stratum II or III), which also plays a critical role when examinees solve spatial tasks. This evidence suggests that executive components of spatial processing may underpin key spatial reasoning abilities. In future work, one approach is to implement controls on general g and other related abilities by assessing participants’ general intelligence and grouping them into distinct cognitive strategy groups.

The other method is to expand the Q-matrix to include additional cognitive process attributes (e.g., cognitive flexibility, working memory) by adding effective task types. In this study, our mixed spatial test only included three task types (MR, OA, and IP). Although three task types represent diverse spatial requirements, they still possibly miss certain attributes. Next, we could incorporate additional task types, such as the paper folding test (Ekstrom and Harman 1976), the perspective taking test (Hegarty and Waller 2004), and the road map test (Mondy and Walker 1965), to enhance the comprehensiveness of the spatial ability assessment and allow for broader construct representation.

## Figures and Tables

**Table 1:** Major Categories and Theories of Spatial Ability Across Key Studies.

Author(s)	Major Categories of Spatial Ability
Linn and Petersen (1985)	Spatial Perception, Mental Rotation, and Spatial Visualization
Lohman et al. (1987)	Spatial Visualization, Spatial Relations, and Spatial Orientation
Carroll (1993)	Fluid Intelligence, Visualization, and Spatial Relations
Hegarty et al. (2010)	Working Memory, Perspective-Taking, and Mental Transformations
Uttal et al. (2013)	Intrinsic vs. Extrinsic, Static vs. Dynamic, and Trainability
Lakin (2024)	Visualization, Rotation, Orientation, Perception, Mechanical Reasoning, and Dynamic Spatial Ability

**Table 2:** Relationship of Test Type and Spatial Ability Attributes, based on Linn and Petersen (1985).

Test	SP ^1^	MR ^2^	SV ^3^
Mental Rotation Test	0	1	0
Object Assembly (WAIS)	1	1	0
Isometric Perspective	1	0	1

Note. ^1^ SP = Spatial Perception; ^2^ MR = Mental Rotation; ^3^ SV = Spatial Visualization.

**Table 3:** Relationship of Test Type and Spatial Ability Attributes, based on Hegarty et al. (2010).

Test	MT ^1^	SV ^2^	SO ^3^	SWM ^4^
Mental Rotation Test	1	0	0	0
Object Assembly (WAIS)	1	1	0	1
Isometric Perspective	0	1	1	0

Note. ^1^ MT = Mental Trans-formation; ^2^ SV = Spatial Visualization; ^3^ SO = Spatial Orientation; ^4^ SWM = Spatial Working Memory.

**Table 4:** Relationship of Test Type and Spatial Ability Attributes, based on Uttal et al. (2013).

Test	Intrinsic ^1^	Extrinsic ^2^	Static ^3^	Dynamic ^4^
Mental Rotation Test	1	0	1	1
Object Assembly (WAIS)	1	1	1	0
Isometric Perspective	1	0	1	0

Note. ^1^ Intrinsic: Spatial processing that focuses on the internal configuration of an object. ^2^ Extrinsic: Spatial processing involving relations between objects or between an object and its environment. ^3^ Static: Involves processing fixed spatial information with no transformation. ^4^ Dynamic: Involves spatial transformation or mental manipulation of objects in space.

**Table 5:** Relationship of Test Type and Spatial Ability Attributes, based on Lakin (2024).

Test	Vi ^1^	Ro ^2^	Or ^3^	Pe ^4^	MR ^5^	DSA ^6^
Mental Rotation Test	0	1	0	1	0	1
Object Assembly(WAIS)	1	1	0	1	1	0
Isometric Perspective	1	0	1	1	0	0

Note. ^1^ Vi = Visualization; ^2^ Ro = Rotation; ^3^ Or = Orientation; ^4^ Pe = Perception; ^5^ MR = Mechanical Reasoning; ^6^ DSA = Dynamic Spatial Ability.

**Table 6:** Model Fit Comparison for CDMs.

Model	logLik	Deviance	AIC	BIC	CAIC	SABIC
DINA	−2327.23	4654.46	4960.46	5389.48	5542.48	4905.72
DINO	−2327.25	4654.49	4960.49	5389.51	5542.51	4905.76
GDINA	−2194.60	4389.21	5427.21	6882.49	7401.49	5241.52
ACDM	−2205.72	4411.43	4921.43	5636.46	5891.46	4830.20
LLM	−2151.60	4303.19	4813.19	5528.22	5783.22	4721.96
RRUM	−2196.93	4393.85	4903.85	5618.88	5873.88	4812.62
HO-GDINA ^1^	−2080.57	4161.13	5097.13	6409.41	6877.41	4929.69
HO-LLM ^2^	−2168.01	4336.01	4744.01	5316.03	5520.03	4671.03

^1^ HO-GDINA = High-order GDINA; ^2^ HO-LLM = High-order LLM.

**Table 7:** Model Fit Comparison for Q-matrices.

Q-Matrix	Att. No. ^5^	Relative Index				Absolute Index		CA ^6^
		AIC	BIC	CAIC	SABIC	RMSEA2	SRMSR	
Q_LP_ ^1^	3	4820.83	5179.74	5307.74	4775.03	0.049	0.097	0.931
Q_H_ ^2^	4	4783.23	5181.41	5323.41	4732.43	0.045	0.092	0.879
Q_U_ ^3^	4	4767.53	5238.60	5406.60	4707.42	0.048	0.087	0.843
Q_LH_ ^4^	6	4744.01	5316.03	5520.03	4671.03	0.045	0.085	0.708

^1^ Q_LP_ = Q-matrix based on Linn and Petersen (1985); ^2^ Q_H_ = Q-matrix based on Hegarty et al. (2010); ^3^ Q_U_ = Q-matrix based on Uttal et al. (2013); ^4^ Q_LH_ = Q-matrix based on Lakin (2024); ^5^ Att. No. = number of attributes; ^6^ CA = Classification Accuracy.

## Data Availability

Available on request through the third author.
